# ‘I’m sure we made it a better study…’: Experiences of adults with intellectual disabilities and parent carers of patient and public involvement in a health research study

**DOI:** 10.1177/1744629517723485

**Published:** 2017-08-16

**Authors:** Carole Beighton, Christina Victor, Iain M Carey, Fay Hosking, Steve DeWilde, Derek G Cook, Paula Manners, Tess Harris

**Affiliations:** St George’s University of London, UK; Brunel University London, UK; St George’s University of London, UK; St George’s University of London, UK; St George’s University of London, UK; St George’s University of London, UK; St George’s University of London, UK; St George’s University of London, UK

**Keywords:** intellectual disabilities, parent carers, PPI, public involvement, participatory inclusive research

## Abstract

Patient and public involvement is considered integral to health research in the United Kingdom; however, studies documenting the involvement of adults with intellectual disabilities and parent carers in health research studies are scarce. Through group interviews, this study explored the perspectives and experiences of a group of adults with intellectual disabilities and a group of parent carers about their collaborative/participatory involvement in a 3-year study which explored the effectiveness of annual health checks for adults with intellectual disabilities. Thematic analysis identified five key themes consistent across both groups; authenticity of participation, working together, generating new outcome measures, dissemination of findings and involvement in future research. Although reported anecdotally rather than originating from the analysis, increased self-confidence is also discussed. The groups’ unique perspectives led to insights not previously considered by the research team which led to important recommendations to inform healthcare practice.

## Background

Within the context of health and social care research, many countries actively engage patient and public involvement (PPI). PPI (also variously referred to as service user or user involvement) is defined as ‘…ways in which patients can draw on their experience and members of the public can apply their priorities to the evaluation, development, organization and delivery of health service’ (Tritter, 2009: 276). It is now accepted that service users should be active participants in their own health and well-being (Morrow et al., 2012), and PPI is premised on the assumption that it will improve the way the research is prioritised, commissioned, undertaken, communicated and used (Brett et al., 2012). This active involvement of service users is seen as adding a unique perspective that can strengthen the quality of the health research (National Institute for Health Research (NIHR), 2015) and ensures that it remains relevant to all those using health and social care services (Oliver et al., 2008). However, it is suggested that the majority of PPI activity in the United Kingdom is concentrated at the lowest levels of involvement, namely, feedback and information sharing (Ocloo and Matthews, 2016; Tritter and McCallum, 2006).

In England, involving patients and the public in research has increasingly become a pre-requisite for funding bodies including the NIHR who funded us to undertake a 3-year retrospective evaluation of the effectiveness of annual health checks in primary care for adults with intellectual disabilities. This health research study used anonymized patient records from a large primary care database of patient records from approximately 400 general practices (GPs) across England (Herrett et al., 2015).

NHS England (2013: 27) contends that everyone should contribute to PPI ‘…especially those who face the greatest health disadvantage and the poorest health outcomes’. Carers in general and people who have intellectual disabilities are known to face disadvantage, have poorer health outcomes and higher mortality than the general population (Carey et al., 2016; NHS England et al., 2016). Their reported contribution to PPI within a health context is limited (Gibbs et al., 2008) and although the core principles of good practice for PPI in research applies to all, the experiences of people who have intellectual disabilities and carers are often overlooked (Repper and Simpson, 2011). There are examples of the active involvement of people with intellectual disabilities in the fields of education, health improvement and social research where PPI is termed ‘inclusive’ research; however, this term is still not widely used (Nind, 2014). Inclusive research is ‘research which includes or involves people with learning disabilities as more than just subjects of research’ (Walmsley and Johnson, 2003: 9) and to be considered inclusive it has five key characteristics (Box 1):

Box 1. Five key characteristics that makes research inclusive.

- The ‘problem’ should be owned by the disabled person
- The research should further the interests of disabled people
- The research should be undertaken collaboratively and involve people with intellectual disabilities throughout the research process
- People with intellectual disabilities should be able to exert some control over processes and outcome
- The research question, process and reports must be accessible to people with intellectual disabilities

Inclusive research embraces a range of research approaches including participatory and emancipatory. In participatory research, researchers work alongside people with learning disabilities as active participants rather than being passive recipients (Kiernan, 1999), a development of the drive towards emancipatory research led by Zarb (1992) and Oliver (1992) which is underpinned by the key principles of empowerment, reciprocity and gain and whereby people with disabilities control the research agenda (Chappell, 2000). The latter approach is less well established within health research although it is clear that people with intellectual disabilities have the right to be consulted about, and be actively involved in research which affects their lives and that there is a strong moral case based on social justice for doing so (Northway, 1998; Stalker, 1998; Szmukler et al., 2011). However, it should be acknowledged that for people who have an intellectual disability, some activities that require a high level of abstraction make it less amenable to emancipatory practice (Walmsley, 2004).

Within the specific health service research context that includes carers, INVOLVE identifies three distinct approaches to PPI, although research studies may demonstrate a combination of these three approaches (NIHR, 2012). These approaches are consultation (asking for views and using them to inform decision-making), collaboration (an active ongoing partnership) and control (professionals are involved by invitation and the design, implementation and dissemination of results are undertaken by the user group) (INVOLVE, 2015). This later type of PPI, control, is redolent of the emancipatory inclusive research approach noted earlier, while the collaboration model approximates to the model of participatory inclusive research.

It is reported that although PPI involvement within health research has increased over past decade, there is still a relatively weak evidence base underpinning it, primarily due to poor reporting (Oliver et al., 2008; Staniszewska and Denegri, 2013) which can limit the potential of learning from practice (Wright et al., 2010). Understanding of the perspectives and experiences of those involved in PPI is also limited (Thompson et al., 2014) especially for those with learning disabilities or parent carers as there is a paucity of literature in this area (Gibbs et al., 2008; Miller et al., 2006) as people with intellectual disabilities can be excluded from being involved in research as their participation can be perceived as being ‘too difficult’ (Aldridge, 2014).

## Aim of study

The main aim of this study was to explore the perspectives and experiences of adults with intellectual disabilities and parent carers of their public and participant involvement in a health research study over a 3-year period.

## Our model of PPI

Although inclusive research is the term used to describe active involvement with people with intellectual disabilities, in recognition of the fact that we equally worked with parent carers, we will use both the terms PPI and inclusive research and collaborative/participatory approaches.

It is argued that the design and intentions of a research study should be developed to be of an emancipatory or control nature (Chappell, 2000). However ‘inclusive research has no fixed formula’ (Ollerton, 2012: 5) and as there is lack of clarity as to the research designs and methods that best fit inclusive health research, it is recommended that the design should be tailored to each specific study (Frankena et al., 2016). Given the nature of our research study, a secondary data analysis of a large GP database, we were unable to implement an emancipatory user-led or control approach with our groups and instead agreed to work together using a collaborative/participatory approach. This approach to PPI has been found to be ‘meaningful’ at lower levels with service user and carer contributions producing ‘powerful influences’ on social work research in the absence of full partnerships or user control (Fleischman, 2010).

It was agreed their involvement would include the following activities:

1. inform the choice of process and outcome measures and/or identify alternate outcome measures to be used,
2. develop ideas for further explanatory analysis for our findings,
3. interpret the findings of the study,
4. disseminate results co-presentation with research staff and
5. Form recommendations for further research and policy.

Two established service user groups known to one member (C.B.) of the research team became involved in September 2013. Established groups with previous experience of involvement in healthcare projects were chosen as opposed to recruiting individuals, because of the well-documented difficulties in recruiting service users with intellectual disabilities, including access problems from the hierarchy of gatekeepers and research governance and ethical issues specifically relating to issues of capacity to consent to participate (Cameron and Murphy, 2006; Goldsmith and Skirton, 2015; Nicholson et al., 2013; Nind, 2008; Tuffrey-Wijne et al., 2008).

One group, ResearchNet, comprised of adults with intellectual disabilities and had been facilitated by two healthcare professionals (including P.M.) for a number of years. One of the researchers (C.B.) had also worked with the group over this time and had built up a relationship with them, negating the need to build up trust prior to commencing the study (Goodley, 1998). These longstanding relationships also substantially minimized the potential for an unequal power relationship between researchers and the group (Dalton and McVilly, 2004; Freedman, 2001). Our other group was a partnership group of parent carers who supported their adult children with intellectual disabilities. All except one mother was unrelated to the members of ResearchNet. We engaged parent carers as a separate group as they bring their own distinct perspective and experiences as it is important to consider their views separately from those they support (Repper and Simpson, 2011).

From the outset, both groups were provided with clear information regarding the aims of the main health research study, the level and scope of involvement and the period of time over which their involvement would be required and for ResearchNet this information was made accessible (NIHR, 2015). Involvement was the same for both groups and due to their other commitments and the set time frame posed by our funding body, six meetings per year over the 3 years were negotiated and scheduled; however, due to the death of the principal researcher in 2015 who facilitated both groups, one less meeting took place for each group. Prior to each meeting, details were sent out regarding the topic area the researchers would like their involvement with, for example, queries which had arisen from the data analyses. Again, information for each session with ResearchNet was presented in an accessible format tailored to each individual’s level of understanding and communication needs consistent with Brooks and Davis (2008: 130) who suggest that for people who have an intellectual disability, information may need to be absorbed over time with understanding reached in ‘the doing’. The ResearchNet facilitators knew each group member and their abilities well and they played a central role in being able to adapt questions for individuals using their skills to prompt some to recall experiences, to draw out responses and to provide support and encouragement to contribute.

## Evaluation of involvement in the health research study

Group interviews were chosen as the best method to elicit the group’s perspectives and experiences of their involvement in the health research study. This method allows for the researcher to ask questions to specific members to ensure that all were able to participate, thereby reducing the possibility of acquiescence bias (Tassé et al., 2005). Group interviews can also help build confidence, empower members, provide for inter-member reinforcement, peer support and validation of views and experiences (Cambridge and McCarthy, 2001; Tassé et al., 2005).

**Figure 1.**
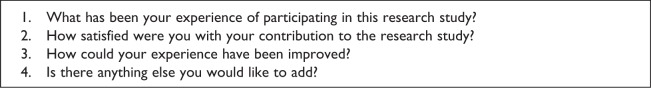
Semi-structured topic guide used for both groups.

Following discussions within the research team, it was decided to use a semi-structured topic guide which contained four open questions with the same guide being used for both groups (Figure 1) with additional probing (as required) to further explore specific responses. These were not co-produced with the groups as we did not want them to be aware of what we were going to ask to prevent pre-determined answers. Although the aim was to ask both groups the same questions, a pragmatic stance was taken in that each question would be broken down, explained or rephrased allowing each individual with intellectual disabilities to make a meaningful contribution.

The group interview for the ResearchNet members was scheduled to take place a week after a conference where the group had co-presented the findings of the health research study in collaboration with the research team so that this would be fresh in their minds. Due to the short attention span of some members of the ResearchNet group, a 1-hour maximum limit was established for the group interview. Prior to commencing each group interview, both groups were assured that there were no right or wrong answers to the questions and that all reflections and personal experiences were valuable. Consent was obtained to participate and for the group interviews to be audio-recorded. They were also reminded of their control over withdrawing and leaving the group at any time without explanation and that it was their choice whether to answer questions or not. In the ResearchNet group, although confidentiality was easily understood, there was difficulty explaining the concept of anonymity, and therefore, it was agreed that the best approach would be for each participant to adopt the name of their favourite singer to hide their identity.

Both group interviews were undertaken in the same environment the meetings for the health research study had taken place over the previous 3 years which allowed for a relaxed, informal environment (Kaehne and O’Connell, 2010). The participants prolonged relationship with each other, the facilitators and the research team allowed for an ‘anti-authoritative and non-hierarchical atmosphere’ (Karnieli-Miller et al., 2009: 280), reducing potential power imbalances and allowing the group to feel safe enough to talk openly about their experiences (Edwards and Holland, 2013). Our plan was to run both group interviews with a facilitator and researcher present. This was achieved for the ResearchNet group, but the carers group was run by the researcher alone because of the last minute unavailability of the second facilitator. Rather than cancel this group interview, reflective and descriptive notes were made of interactions and noticings immediately afterwards by the researcher. Stalker (1998) identified the importance of providing refreshments for people with intellectual disabilities while interviewing, and these were provided for both groups at all meetings and the group interviews.

## Analyses

The audio recordings of the group interviews were transcribed verbatim by an external company. During the interview with ResearchNet, it became too complicated and disruptive to keep reminding participants to say their singer pseudonym prior to answering a question; therefore, the researcher who undertook the group interview assigned participant numbers to the transcript by listening to the audio file. The transcripts were also checked for accuracy at this time. Checking of transcripts and coding was guided by thematic analysis (Braun and Clarke, 2013) undertaken independently by the group facilitator and the researcher who had undertaken both group interviews. The findings, any queries or areas of disagreement were discussed between them and also with the wider research team to ensure consensus and to increase reliability. The aim of the analysis was to report and describe thematic groupings as they appeared within the data set therefore staying ‘closer’ to the data obtained (Pope et al. 2006; Neergaard et al. 2009: 2). The thematic analysis was conducted without the assistance of both groups so not to introduce bias but in order to increase validity and credibility, the initial findings were sent to both groups for member checking.

It is argued that to enable independent validation of data, findings should also include direct quotations to ‘…demonstrate the mutual engagement of participants and the clear expression of coherent views and opinions’ (Kaehne and O’Connell, 2010). Therefore, we have deliberately included some longer quotations to provide insight into how questions were adapted to ensure they were understood by individuals with intellectual disabilities and to allow their own voices to accurately reflect their intended meanings and not we, as researchers bringing our own interpretations to their words (Bogdan and Biklen, 1998)

## Findings

The group interviews were undertaken in June and August 2016, each lasting around 1 hour. The four parent carers were females and the ResearchNet group contained five adults who had mild to moderate intellectual disabilities and contained two females and three males aged between 27 and 40 years. Both groups had long and varied experiences of using health services.

For both groups, five similar key themes were identified from the thematic analysis: authenticity of participation, partnership working, generating new outcome measures, dissemination of findings and future involvement in research. Although not identified as a theme following the analysis, it was noted anecdotally that in both groups their self-confidence had increased during the process of their involvement and this will also be discussed. These key themes will now be presented along with illustrative quotes. Quotes from participants are indicated using italics and quotation marks and those from the facilitator/researcher by bold print. The parent carers’ quotes are denoted by ‘PC’ and the adults with intellectual disabilities by ‘P’.

## Authenticity of participation

Both groups perceived their participation to be authentic and not tokenistic, with their contribution respected and seen as valid and important by the research team. They described feeling listened to and that there was a genuine interest in their experiences, leaving them feeling confident that their views would be carried forward. With the parent carers, this positive experience had stemmed from when they were initially consulted to see if they would be interested in participating. They recalled the aim and the methods of the research study and the ways in which they could provide input into the study were set out very clearly to them by the principal investigator at the beginning. Both groups agreed unanimously that this and the subsequent meetings were a positive experience for them:

I genuinely felt, and I’ve said this to various people, but this wasn’t just a tick box exercise, ooh yes, I’ve consulted carers, it was a genuine…let’s see how you can get involved and I’d like to incorporate your ideas in it, so it did feel like genuine involvement which was great. (PC3)

‘We, we are actually being listened to and taken note of. Our opinions counted taken seriously’. (P3)

The parents felt that their suggestions and experience had made a strong contribution to the study, recounting how important it was to them to have been able to address issues of relevance to them and not just contribute to what the researchers wanted to study. This illustrates that our parent carers clearly aspired to a collaborative/participatory rather than a consultative model of engagement. They described how when suggestions were made by them, none were ever ‘shrugged off’ or dismissed as irrelevant:

…one of the main things I got from this was that he was interested in the project from our point of view, what was useful, helpful and interesting to us as carers, not just the scientific community, and actually trying to get useful information from us for the studies, not just interesting scientific information…you know, looking at the mortality rates, looking at how GPs interact and check-ups and all of those/that’s the things I need to know about as her carer. (PC1)

## Working together

Following each phase of the secondary data analysis for the health research study, both groups participated in the interpretation and offered possible explanations for the findings which is where their personal experience and contributions were invaluable. For example, one analyses highlighted that some adults with intellectual disabilities were not having diagnostic blood tests or flu vaccinations at their doctors surgery. Both groups provided insight into the fact that from personal experience it was most likely because of a severe needle phobia and not that it wasn’t offered by their GPs. One parent carer recalled a similar discussion about accident and emergency admission rates within one meeting:

We spent a lot of time trying to work out why accident and emergency admission rates were higher for particular conditions and whether it was that people tended to play safe and panic slightly more because they couldn’t get as clear information from the person they cared for, whether they lived at home, or in residential care, as to how serious the problem was and hence were more likely to go A&E particularly out of hours…I remember a lot of discussion about that. (PC4)

## Generating new outcome measures

The research team had identified key outcome measures at the beginning of the health research study based upon the available GP database. In discussion with both groups, alternative outcome measures important to each of the groups emerged, which initiated further exploration by the research team. Two of these alternative outcome measures were mentioned by both groups independently and identified as important both at a practical level and for improving the quality of GP consultations at the surgeries: (i) longer GP appointments and (ii) seeing the same GP.

…we were talking about the variation in GP practices and how difficult some people found it to see the same GP and how incredibly important that continuity was but it didn’t always happen…people should look in to this and should think carefully about providing both possibly longer appointments more often but also that continuity of care and how to do it. (PC3)

‘Because you can get everything in on a double appointment. You can’t just get everything in on one.’ (P2)

‘I have different doctors. I have different doctors and they’re just like/I don’t have the same doctor/ and it’s not good…I want routine. I want the same doctor but all/all jump around and/Jump around…. they know who you are…. (P4)

Although the parent carers were pleased that their suggestions were taken on board, they also expressed frustration that some of their suggestions could not be explored further due to the limitations of the information held on the GP database, however this also contributed to them gaining a greater insight into the research process:

…the main thing we would have liked to have seen was more analysis by where people lived and who they lived with, whether they lived with their family, whether they lived independently, whether they lived in a residential care home, supported living, because we felt that that might explain quite a lot of the variations in things like umm how many people had health checks, how many people were taken to A&E etc…. (PC2)

There’s frustration of the data set and being able to pull out the kind of information we want answers to, but that wasn’t something that was wrong with the study. (PC4)

## Dissemination of health study results

The results of the health research study were disseminated at conferences arranged by both groups and although the research team presented at a local MENCAP (a leading charity for people with intellectual disabilities) conference organized by the parent carers, ResearchNet co-presented with the researchers at their own conference that was attended by around 100 people from around Southern England. Although the study findings had already been produced in an accessible format for them, the method of presenting findings had to be adapted too and involved playing a game with the audience as to whether the medical condition was ‘higher or lower’ in a person with intellectual disabilities than in the general population. Some of the adults with intellectual disabilities had never been to a conference before and were initially scared, but the experience for them was overwhelmingly positive as one recounted:

‘Really good. I just want to do more conferences now. Oh I wonder if we’ll be going up and down the country to do conferences?’ (P3)

In addition to the conference, the parent carers also shared ideas for further dissemination to others as they thought it would be useful for increasing knowledge and improving services:

I don’t know how much has been disseminated to paid carers/ You’ve disseminated it to health staff, you’ve disseminated it to parents, but I just wonder how much of it has been disseminated to paid carers who might then flag up/it might then flag up for them areas that they need…I’m thinking of things like the learning disability home managers but also I’m sort of thinking to managers of services. Perhaps we should do that…. (PC4)

## Future involvement in research

There was overwhelming agreement from both groups that the experience of being involved in the health research study was positive and that they would be open to being involved in future studies, as this parent summed up:

Definitely. I would definitely work with this team from St Georges again as I know that they are serious about what they are doing. You know that they are serious about involving parents and they have listened to us. (PC2)

All but one ResearchNet member, who was hesitant, said they would definitely do it again if the possibility arose, as this exchange shows:

  **‘So if the team came back and asked you if you wanted to be involved in more research like this with them, what would you say?’**

P2: Be careful what the research is

  **‘Very good point actually. Is that everybody would like to do it again or not?’**

P2: Not really.

  **‘Not really? Is there anything that might put you off?’**

P2: Uhh/to talk about uhh / uhh / some things that people do is bad, I don’t really fancy talking about it.

  **‘Right, so just so that I’m clear, is that like things that people do to other people?’**

P2: Yes

  **‘Because they’re tricky to talk about aren’t they?’**

P2: I don’t like talking about them

  **‘Sure, yes, I think that’s a really important point isn’t it?’**

P2: Yes

This quote highlights how researchers should be aware that any interview situation has the potential to cause distress, especially when asking participants to disclose personal experiences (Edwards and Holland, 2013). The experience of the group facilitator in knowing the individual well was key to understanding this interaction as they were aware that the participant was referring to a previous upsetting interview experience and skilfully deflected this. This also highlights that the group were comfortable speaking up and acquiescence was not considered to be a potential problem within this group.

The parent carers also provided suggestions on what they thought might be important for researchers to focus on for future studies to improve the overall health and well-being for people with intellectual disabilities based on their ‘real-world’ needs. These included wanting to explore variation in the quality of annual health checks (i.e. using the omission of a health action plan as a proxy indicator of how good the check had been) and exploring the views of paid carers/support workers. There was overwhelming support from all to improve communication systems:

Communication. Everything comes back to communication…how to set up the best possible communication system between family carers, person with the learning disability and all the medical professionals they come in to contact with, to ensure the optimal treatment and who would perform what role within that system. (PC3)

## Increased confidence

Although not identified as a theme from the analysis, it was noted anecdotally by the research team and others that both groups had grown in confidence over the course of their involvement. Similarly, from the previous theme the parent carers had clearly considered other areas that they felt could be researched and this experience had left them wanting to be involved in future studies. However, they were emphatic that they would like to be involved from inception of any research study, taking more of a ‘control’ approach. One member of ResearchNet recalled their participation at the conference when prompted:

  **‘You were telling me earlier that your sister was surprised at how far you’d come?’**

P3: ‘Yes. She was really surprised’

  **‘What was she surprised about?’**

P3: ‘That I could talk to so many people’

  **‘Do you think it has given you more confidence?’**

P3: ‘Yes, I want to do more. Yes. Yes, and other people, she was amazed about and how they come through too…’

The group continued to enthusiastically discuss their contribution to the research; however, it was apparent that they were also very mindful of other’s expectations of them:

P2: I’ve loved doing this but others don’t think we can you know

P5: True

P1: So true

  **‘Why?’**

P2: Because of our learning disability

P5: Yes.

P3: Yes, that’s most people’s opinion.

P2: They don’t understand.

P1: They assume the worst, if you know what I mean, it’s like, oh, this person’s got a learning disability, don’t think they can do that. They judge you before they even know you.

## Discussion

The aim of this study was to explore the perspectives and experiences of adults with intellectual disabilities and parent carers of their patient and participant involvement in a health research study over a 3-year period. We actively involved two groups that are traditionally excluded as they are ‘hard to reach’ (Ocloo and Matthews, 2016).

We acknowledge from the outset that the emancipatory/control approach remains the gold standard (NIHR, 2015; Walmsley, 2001). However, the nature of our health research study, a secondary analysis of a large GP data set, which required considerable technical expertise to undertake the analysis, precluded a truly emancipatory/control inclusive approach. However, we were committed to using a collaborative/participatory approach to working with both groups rather than just consulting with them, thus allowing their involvement to be meaningful and therefore the research findings were still informed and influenced by people with learning disabilities and parent carers themselves (Durell, 2016).

We chose to work with established groups although it is argued that self-advocacy groups can be ‘over-researched’ and can increase the likelihood of obtaining previously sanctioned and rehearsed answers (Kaehne and O’Connell, 2010: 141). However, initial conversations had established that neither group had participated in research in the area being explored and a systematic review found no evidence supporting that any particular method of patient engagement is better (Domecq et al., 2014). Working with established groups meant that members were relaxed and comfortable with each other in the meetings and the group interviews and they were not concerned about voicing their own opinions; and as with other studies, established friendships and knowledge of each other’s situations meant that contributions were encouraged and validated by each other (Barr et al., 2003; Tyrer et al., 2016).

Consistent with previous inclusive qualitative studies, we found that additional time was required both for forward planning and to allow for effective communication to take place within the ResearchNet group (Nind, 2008; O’Brien et al., 2014; Whitehurst, 2007). For example, for each health research meeting, both groups had to commence with a recap and summary of what had been covered in the previous meeting for new members attending or for others who had missed the last meeting. While this was quite brief with the parent carers, with the ResearchNet group, this recap could take up to half of the allocated 1-hour meeting time.

As with the meetings for the health research study, the ResearchNet members required support during the group interviews to remember their contribution to the study and additional time was required for them to process the questions and formulate their responses. The role of the ResearchNet facilitator was invaluable throughout as their relationship with, and knowledge of each of the group members and their skill in adapting the questions, ensuring all were given an opportunity to contribute. Bollard (2003: 162) reports that with a skilled facilitator, participants are able to take turns in contributing, enabling participants to ‘collectivize’ their personal experiences. However, it is acknowledged that this support may distort views through the nature and phrasing of questions and the limited vocabulary available to the person with intellectual disabilities to express their views which can affect interpretation of these views resulting in poor validity/credibility of the findings (Whitehurst, 2007). Although we cannot say this was completely mitigated, we addressed this through triangulation (Patton, 2015) as we were able to increase validity through cross verification of findings between groups as evidenced by the similarity in themes identified from the thematic analyses.

Although the researchers undertook the analyses for the health research study, both groups equally provided expertise in interpreting the data and generated new ideas and outcome measures which offered a different perspective on findings and helped the researchers develop their understanding rather than just focusing from an academic perspective (Omeni et al., 2014; Repper and Simpson, 2011; Ross et al., 2005). During this process, both groups independently suggested exploring two factors which were reflective of their first-hand experiences and concerns and which had not previously been considered by the researchers, GP consultation length and seeing the same GP at each consultation. These suggestions encouraged further investigation of the data set and led to the inclusion of two new outcome measures (Brett et al., 2012). These became key measures of healthcare effectiveness and were important additions to the study which strengthened our published research and formed the basis of recommendations in one publication (Carey et al., 2016). This highlights the importance of PPI in helping to shape research priorities to close the gap between research agendas and the ‘real-life’ needs of patients and carers (Chalmers et al., 2014; Snow et al., 2015).

Other studies have reported barriers to PPI which includes capacity to consent by the people with intellectual disabilities, patients/service users having a different emphasis to those chosen by clinical academic researchers or logistics such as the extra time required (Domecq et al., 2014). We were privileged to be working with two experienced groups who had both been advisors to and involved in a number of other projects and had clarity over their role within this research study. In addition, we had a learning disability nurse in the team who had previously worked with ResearchNet and the group facilitator who knew the group well and therefore we were knowledgeable from the outset that we would need to include extra time and be flexible and pragmatic in our methodological approach. For example, it was recognized prior to starting the study that caring commitments and the reliance on others to accompany some adults with intellectual disabilities to the meetings could influence participation levels and also some of the practical challenges of undertaking group interviews with the ResearchNet members, such as accessibility of information were pre-empted. In addition, being flexible and having the experience of being able to respond to specific challenges ‘in the moment’ has been found to enhance the quality of inclusive research (Miller et al., 2006; Nind and Vinha, 2014: 108).

Although this helped to pre-empt some of the difficulties, tensions and ‘inevitable’ conflicts of interests which other researchers have encountered with PPI (Seddon et al., 2004), there was one area that caused debate. The parents did express frustration as they wanted to explore some aspects further. We accommodated these requests as best we could within the scope of the research and the data available, but this was not possible due to limitations with the database. However, this example did serve to help increase the parents group understanding of what could (and could not) be achieved using different research designs and secondary data.

Both groups were active in disseminating the results of the health study which has been reported to increase the likelihood of people acting on the findings (Staley, 2009). Although the publications in peer-reviewed journals were not co-authored (Smith et al., 2008), both groups had their input acknowledged (Carey et al., 2016; Carey et al., 2016; Hosking et al., 2016) and two parent carers agreed to provide input into writing the plain English summary for the final funding report. Since all publications had open access both groups could directly access them and share the findings. It is suggested that involving service users in the dissemination of research findings can have a powerful impact (Brett et al., 2012; Smith et al., 2008) and both groups organised conferences which not only included their own networks but strategic people such as heads of service and commissioners (Rowe, 2006). At their conference, the ResearchNet members co-presented the health study findings with the researchers. In some PPI studies where service users have intellectual disabilities or mental health problems, difficulties in finding a way to finish this relationship have been reported (Nicholls et al., 2008); however, in addition to celebrating our achievements together it was clear to all that the conferences also marked the end of the health research study for everyone involved.

Although not described by themselves, improved self-esteem, increased self-confidence and empowerment from their involvement has been reported across a range of PPI and inclusive research studies (Gilbert, 2004; NIHR, 2015; Omeni et al., 2014). Increased self-confidence was noted anecdotally in both groups demonstrating the value of this work. This was evidenced by the ResearchNet group who decided themselves they wanted to develop a training package for health-care professionals about the health of people with intellectual disabilities using findings from the health research study. They also co-presented the findings of the health research study and their new training package at the conference. However, the perception of people with intellectual disabilities and their capacity to contribute meaningfully to communities by others who do not have an intellectual disability was raised by ResearchNet during the group interview (Bates and Davis, 2004). People with disabilities in general are disempowered by society (Beresford, 2002) and therefore working in partnership together with the research team will strengthen their position and help bridge social capital at an individual level not only by building up their skills, knowledge and personal empowerment but by also connecting them across social divides which can help contribute towards increasing social inclusion (Bates and Davis, 2004; Bollard, 2009; Pavey, 2006; Portes, 2000). Similarly, increased self-confidence was also evident for the parent carers as they spoke about wanting to be actively involved in research from the beginning, being able to influence the design phase and to be involved through to dissemination. This suggests that as with ResearchNet, their confidence had increased and they were aspiring towards a control model of PPI.

While we have reported the process of the groups involvement, as with other studies, we are unable to quantify the outcome that their involvement made in the research process to date (Omeni et al., 2014; Snape et al., 2014; Staley, 2009). We can however define the ‘impact’ that their involvement has had (Mockford et al., 2012). Their involvement improved the quality and relevance of the research into annual health checks for adults with intellectual disabilities as without their input, two important findings may not have been considered by the researchers. These personal insights have provided a stronger evidence base that has been used to inform healthcare policy and practice through publication. This has been noted in our final report to funders.

The NIHR’s vision for PPI is that by 2025 the public, researchers, health professionals, NHS staff and others will be ‘equal partners’ in creating knowledge, with service users acting as co-researchers working alongside academic and professional colleagues during the course of research studies (2015: 9). Although due to some study designs such as ours, it will not always be possible to have this equal partnership, we would argue from our experience that PPI at a collaborative/participatory level and not only a control/emancipatory level can generate genuine partnerships. Both groups described their involvement as being a positive experience and that they had enjoyed working collaboratively with the team. They genuinely felt that this experience was meaningful for them and their contributions had been valued. Both felt that their input had enhanced the findings, ‘…making it a better study’.

Echoing other PPI studies, all the parent carers agreed they had gained a greater understanding of the research process from their experience (Minogue et al., 2005; INVOLVE, 2015). Both groups valued the opportunity to bring their own real-life experiences to the health research study (Tyrer et al., 2016) and although tokenism is reported as being common in relation to PPI (Snape et al., 2014) our groups perceived their participation to be genuine and described being listened to and their input taken seriously (Omeni et al., 2014). We feel that this demonstrates an equalization of power as opposed to researchers holding the power which has been recognized as a barrier to inclusive research (Chappell, 2000). We have also described our methodology and in addition to our qualitative findings we feel these demonstrate the five key characteristics that must be exhibited for research to be considered inclusive (Walmsley and Johnson, 2003). In addition, we would argue that their involvement was emancipatory as it met the key principles of empowerment, reciprocity and gain for all those involved (Fleischman, 2010; Martin, 2015).

## Implications for practice

The involvement of people with intellectual disabilities and parent carers in PPI and inclusive research, especially research which is of a quantitative nature is often overlooked as it is often seen as being too difficult to facilitate. We have described our methodological approach which includes the practical and organizational strategies we adopted to working with these two groups and which provides insights for other health researchers. As with our study, challenging assumptions and using inclusive methodologies to facilitate active involvement will provide a stronger evidence base to inform healthcare for people with intellectual disabilities and their families.

## Study Strengths

An important strength of this study is that it addresses a paucity of knowledge and strengthens the evidence base by reporting the experiences of PPI in health research of a quantitative nature from the perspective of two groups who are often excluded. We recruited established groups with previous experience of PPI and both the facilitator and researcher undertaking the group interview with ResearchNet members were well known to the group. It could be argued that the facilitators’ and researcher’s prior knowledge of participants may have introduced bias by influencing the direction of discussions or introducing response bias. However, we judged that the group of adults with intellectual disabilities would not participate (either refuse to come to the group or not say anything) with a researcher they did not know. We believe that this familiarity was a strength, as it allowed the individuals to be more open and honest than they might have otherwise been and the accounts more reflective of their true feelings towards their participation.

This article was circulated to and discussed with the parent group to review for accuracy prior to submission and minor amendments made, which increases the authenticity and trustworthiness of the findings. We were not able to meet again with the adults with intellectual disabilities as the group had disbanded for 1 year; however, the group facilitator (P.M.) verified that it accurately reflected what had been discussed in the interview on their behalf.

## Study Weaknesses

Although between four and eight parent carers had regularly attended each group meeting, only four attended the group interview. Similarly, although 6–10 adults with intellectual disabilities regularly attended the meetings, only 5 attended the group interview. However, those who participated were core members who had attended most of the meetings over the study period and it was felt that this number was acceptable. Despite a small number attending, rich data were collected on their personal experiences.

The decision to limit the group interview with the ResearchNet members to one hour meant that some of the depth of probing for some of the answers could have been compromised. We did consider a second group interview but the timing would have meant it would be scheduled after the summer break and it was felt that this would not be helpful for recall of their involvement. A methodological limitation was that the ResearchNet members who participated included those with a mild and moderate degree of intellectual disability, and their views may not be representative of the views of those with severe or profound intellectual disabilities.

The principal investigator who was involved with both groups died 2 years into the study. This may have contributed to a positive reporting/recall bias as he was popular with both groups. Although he attended the parent carer meetings alone, the parent carers’ accounts were consistent with the contemporaneous notes that he had kept of these meetings. The accounts were also consistent with those voiced by ResearchNet in their meetings attended by him and one of the researchers (C.B.) prior to his death.

## Conclusion

Qualitative exploration found that both groups were very positive about their involvement in this health research study. Due to the study design, their involvement was collaborative/participatory and not one of control/emancipatory; however, the five key characteristics that must be exhibited for research to be considered inclusive were met. Both groups perceived their involvement to have been authentic, not tokenistic with their input adding value and credibility to the findings and were keen to be involved in future research studies. Adopting a pragmatic approach to the research methods enabled both groups to be active participants enabling us as researchers to gain greater insight into their unique perspectives which might not be considered otherwise. Their involvement contributed to changes to the design of the study in terms of choice of outcomes, examination of potential modifying factors and help in interpreting and disseminating findings. As a result of their personal insights, two new outcomes were identified that led to important recommendations to inform healthcare policy and practice through publications. Their participation also increased self-confidence and social capital for both groups and provides a strong justification for actively involving both adults with intellectual disabilities and parent carers in future health research to guide improvement in both the health and lives of people with intellectual disabilities.

## References

[bibr1-1744629517723485] Aldridge J (2014) Working with vulnerable groups in social research: dilemmas by default and design. Qualitative Research 14(1): 112–130.

[bibr2-1744629517723485] Barr O McConkey R McConaghie J (2003) Views of people with learning difficulties about current and future accommodation: the use of focus groups to promote discussion. Disability & Society 18(5): 577–597.

[bibr3-1744629517723485] Bates P Davis F (2004) Social capital, social inclusion and services for people with learning disabilities. Disability & Society 19(3): 195–207.

[bibr4-1744629517723485] Beresford P (2002) User involvement in research and evaluation: liberation or regulation? Social Policy and Society 1(2): 95–105.

[bibr5-1744629517723485] Bogdan R Biklen SK (1998) Qualitative Research for Education: An Introduction to Theory and Method. Boston, MA: Allyn and Bacon.

[bibr6-1744629517723485] Bollard M (2003) Going to the doctor. Journal of Learning Disabilities 7(2): 156–164.

[bibr7-1744629517723485] Bollard M (2009) A review and critique In: Bollard M (ed) Intellectual Disability and Social Inclusion: A Critical Review, London: Churchill Livingstone, pp. 5–18.

[bibr8-1744629517723485] Braun V Clarke V (2013) Successful Qualitative Research. A Practical Guide for Beginners. London: SAGE.

[bibr9-1744629517723485] Brett J Staniszewska S Mockford C (2012) Mapping the impact of patient and public involvement on health and social care research: a systematic review. Health Expectations 17(5): 637–650. 22809132 10.1111/j.1369-7625.2012.00795.x PMC5060910

[bibr10-1744629517723485] Brooks M Davis S (2008) Pathways to participatory research in developing a tool to measure feelings. British Journal of Learning Disabilities 36: 128–133.

[bibr11-1744629517723485] Cambridge P McCarthy M (2001) User focus groups and best value in services for people with learning disabilities. Health & Social Care in the Community 9(6): 476–489. 11846827 10.1046/j.0966-0410.2001.00328.x

[bibr12-1744629517723485] Cameron L Murphy J (2006) Obtaining consent to participate in research: the issues involved in including people with a range of learning and communication disabilities. British Journal of Learning Disabilities 35: 113–120.

[bibr13-1744629517723485] Carey I Hosking F Harris T (2016a) Do health checks for adults with intellectual disability reduce emergency hospital admissions? evaluation of a natural experiment. Journal of Epidemiology and Community Health: jech-2016-207557. 10.1136/jech-2016-207557 PMC5256310 27312249

[bibr14-1744629517723485] Carey I Shah S Hosking F (2016b) Health characteristics and consultation patterns of people with intellectual disability. British Journal of General Practice 66(645): e264–270. 26906630 10.3399/bjgp16X684301 PMC4809710

[bibr15-1744629517723485] Chalmers I Bracken MB Djulbegovic B (2014) How to increase value and reduce waste when research priorities are set. The Lancet 383(9912): 156–165. 10.1016/S0140-6736(13)62229-1 24411644

[bibr16-1744629517723485] Chappell A (2000) The emergence of participatory methodology in learning disability research: Understanding th. British Journal of Learning Disabilities 28(1): 38–43.

[bibr17-1744629517723485] Dalton AJ McVilly KR (2004) Ethics guidelines for international, multicenter research involving people with intellectual disabilities. Journal of Policy and Practice in Intellectual Disabilities 1(2): 57–70.

[bibr18-1744629517723485] Domecq JP Prutsky G Elraiyah T (2014) Patient engagement in research: a systematic review. BMC Health Services Research 14(1): 89. 24568690 10.1186/1472-6963-14-89 PMC3938901

[bibr19-1744629517723485] Durell S (2016) ‘Welcome to the real world’ inclusive research with people with learning disabilities: A doctoral journey. The Qualitative Report 21(12): 2308–2330.

[bibr20-1744629517723485] Edwards R Holland J (2013) What is Qualitative Interviewing? ‘What is?’ Research Methods Series. London, UK: Bloomsbury Academic.

[bibr21-1744629517723485] Fleischman P (2010) Mental health service user/survivor research In: Webber M Nathan J (eds) Reflective Practice in Mental Health. Advanced Psychosocial Practice with Children, Adolescents and Adults, London, UK: Jessica Kingsley, pp. 82–99.

[bibr23-1744629517723485] Frankena TK Naaldenberg J Cardol M (2016) Exploring academics’ views on designs, methods, characteristics and outcomes of inclusive health research with people with intellectual disabilities: a modified Delphi study. BMJ Open 6(8): e011861. 10.1136/bmjopen-2016-011861 PMC5013349 27540101

[bibr24-1744629517723485] Freedman RI (2001) Ethical challenges in the conduct of research involving persons with mental retardation. Mental Retardation 39: 130–141. 11340962 10.1352/0047-6765(2001)039<0130:ECITCO>2.0.CO;2

[bibr25-1744629517723485] Gibbs SM Brown MJ Muir WJ (2008) The experiences of adults with intellectual disabilities and their carers in general hospitals: a focus group study. Journal of Intellectual Disability Research 52(December): 1061–1077. 18466292 10.1111/j.1365-2788.2008.01057.x

[bibr26-1744629517723485] Gilbert T (2004) Involving people with learning disabilities in research: issues and possibilities correspondence. Health & Social Care in the Community 12(4): 298–308. 15272885 10.1111/j.1365-2524.2004.00499.x

[bibr27-1744629517723485] Goldsmith L Skirton H (2015) Research involving people with a learning disability – methodological challenges and ethical considerations. Journal of Research in Nursing 20(6): 435–446.

[bibr28-1744629517723485] Goodley D (1998) Stories about writing stories In: Clough P Barton L (eds) Articulating with Difficulty: Research Voices in Special Education, London: Paul Chapman, pp. 113–127.

[bibr29-1744629517723485] Herrett E Gallagher AM Bhaskaran K (2015) Data resource profile: clinical practice research datalink (CPRD). International Journal of Epidemiology 44(3): 827–836. 26050254 10.1093/ije/dyv098 PMC4521131

[bibr30-1744629517723485] Hosking F Carey I Shah S (2016) Mortality among adults with intellectual disability in england: comparisons with the general population. American Journal of Public Health 106(8): 1483–1490. 27310347 10.2105/AJPH.2016.303240 PMC4940652

[bibr82-1744629517723485] INVOLVE (2015) Public Involvement in Research: Values and Principles Framework. Eastleigh, UK: INVOLVE.

[bibr31-1744629517723485] Kaehne A O’Connell C (2010) Focus groups with people with learning disabilities. Journal of Intellectual Disabilities: JOID 14(2): 133–145. 20930023 10.1177/1744629510381939

[bibr32-1744629517723485] Karnieli-Miller O Strier R Pessach L (2009) Power relations in qualitative research. Qualitative Health Research 19(2): 279–289. 19150890 10.1177/1049732308329306

[bibr33-1744629517723485] Kiernan C (1999) Participation in research by people with learning difficulties: origins and issues. British Journal of Learning Disabilities 2: 43–47.

[bibr34-1744629517723485] Martin JA (2015) Research with adults with asperger’s syndrome—participatory or emancipatory research? Qualitative Social Work 14(2): 209–223.

[bibr35-1744629517723485] Miller E Cook A Alexander H (2006) Challenges and strategies in collaborative working with service user researchers: reflections from the academic researcher. Research, Policy and Planning 24(July): 197–208.

[bibr36-1744629517723485] Minogue V Boness J Brown A (2005) The impact of service user involvement in research. International Journal of Health Care Quality Assurance i 18(2): 103–112. 10.1108/09526860510588133 15974504

[bibr83-1744629517723485] Mockford C Staniszewska S Griffiths F (2012) TAQ 10: The impact of patient and public involvement on UK NHS healthcare: a systematic review. International Journal for Quality in Healthcare 24(1): 28–38. 10.1093/intqhc/mzr066 22109631

[bibr84-1744629517723485] Morrow E Boaz A Brearly S (2012) Handbook of Service User Involvement in Nursing and Healthcare Research. Chichester: Wiley-Blackwell.

[bibr37-1744629517723485] Neergaard MA Olesen F Andersen RS (2009) Qualitative description – the poor cousin of health research? BMC Medical Research Methodology 9(April 2016): 52. 19607668 10.1186/1471-2288-9-52 PMC2717117

[bibr38-1744629517723485] NHS England (2013) Transforming Participation in Health and Care. London: NHS England.

[bibr39-1744629517723485] NHS England, Age UK, Carers Trust, et al. (2016) A Practical Guide to Healthy Caring. London: NHS England.

[bibr85-1744629517723485] Nicholls V Wright S Waters R (2008) Surviving User-led Research: Reflections on Supporting User-led Projects. London: Mental Health Foundation.

[bibr40-1744629517723485] Nicholson L Colyer M Cooper SA (2013) Recruitment to intellectual disability research: A qualitative study. Journal of Intellectual Disability Research 57(7): 647–656. 22672134 10.1111/j.1365-2788.2012.01573.x

[bibr41-1744629517723485] NIHR (2012) Briefing notes for researchers: public involvement in NHS, public health and social care research. Eastleigh, UK: INVOLVE.

[bibr42-1744629517723485] NIHR (2015) Going the extra mile: improving the nation’s health and wellbeing through public involvement in research.

[bibr44-1744629517723485] Nind M (2008) Conducting Qualitative Research with People with Learning, Communication and Other Disabilities: Methodological Challenges. Southampton, UK: Project report: National Centre for Research Methods.

[bibr45-1744629517723485] Nind M (2014) Inclusive research and inclusive education: why connecting them makes sense for teachers’ and learners’ democratic development of education. Cambridge Journal of Education 44(4): 525–540.

[bibr46-1744629517723485] Nind M Vinha H (2014) Doing research inclusively: bridges to multiple possibilities in inclusive research. British Journal of Learning Disabilities 42(2): 102–109.

[bibr47-1744629517723485] Northway R (1998) Engaging in participatory research: some personal reflections. Journal of Learning Disabilities for Nursing, Health and Social Care 2: 144–149.

[bibr48-1744629517723485] O’Brien P Mcconkey R García-Iriarte E (2014) Co-researching with people who have intellectual disabilities: insights from a National Survey. Journal of Applied Research in Intellectual Disabilities 27(1): 65–75. 24376031 10.1111/jar.12074

[bibr49-1744629517723485] Ocloo J Matthews R (2016) From tokenism to empowerment: progressing patient and public involvement in healthcare improvement. BMJ Quality and Safety 1–7. 10.1136/bmjqs-2015-004839 PMC4975844 26993640

[bibr50-1744629517723485] Oliver M (1992) Changing the social relations of research production? Disability, Handicap and Society 17(2): 101–115.

[bibr51-1744629517723485] Oliver SR Rees RW Clarke-Jones L (2008) A multidimensional conceptual framework for analysing public involvement in health services research. Health Expectations 11(1): 72–84. 18275404 10.1111/j.1369-7625.2007.00476.x PMC5060424

[bibr52-1744629517723485] Ollerton J (2012) IPAR, an inclusive disability research methodology with accessible analytical tools. International Practice Development Journal 2(2): 1–20.

[bibr53-1744629517723485] Omeni E Barnes M MacDonald D (2014) Service user involvement: impact and participation: a survey of service user and staff perspectives. BMC Health Services Research 14(1): 491. 25344210 10.1186/s12913-014-0491-7 PMC4212124

[bibr54-1744629517723485] Patton M (2015) Analysis, interpretation and reporting, 4th ed In: Patton M (ed) Qualitative Research and Evaluation Methods, Thousand Oaks: CA: SAGE, pp. 520–651.

[bibr55-1744629517723485] Pavey B (2006) Human capital, social capital, enterpreneurship and disability: an examination of some educational trends in the UK. Disability and Society 21(3): 217–229.

[bibr56-1744629517723485] Pope C Ziebland S Mays N (2006) Analysing qualitative data, 3 rd ed In: Pope C Mays N (eds) Qualitative Research in Health Care, Oxford: Blackwell Publishing Ltd, pp. 63–81.

[bibr57-1744629517723485] Portes A (2000) The two meanings of social capital. Sociological Forum 15: 1–11.

[bibr58-1744629517723485] Repper J Simpson A (2011) Good Practice Guidance for Involving Carers, Family Members and Close Friends of Service Users in Research. London, UK: Mental Health Research Network.

[bibr59-1744629517723485] Ross F Donovan S Brearley S (2005) Involving older people in research: methodological issues. Health & Social Care in the Community 13(3): 268–275. 15819748 10.1111/j.1365-2524.2005.00560.x

[bibr60-1744629517723485] Rowe A (2006) The effect of involvement in participatory research on parent researchers in a Sure Start programme. Health & Social Care in the Community 14(6): 465–473. 17059488 10.1111/j.1365-2524.2006.00632.x

[bibr61-1744629517723485] Seddon T Billett S Clemans A (2004) Politics of social partnerships: a framework for theorising. Journal of Education Policy 19(2): 123–142.

[bibr62-1744629517723485] Smith E Ross F Donovan S (2008) Service user involvement in nursing, midwifery and health visiting research: a review of evidence and practice. International Journal of Nursing Studies 45(2): 298–315. 17161402 10.1016/j.ijnurstu.2006.09.010

[bibr63-1744629517723485] Snape D Kirkham J Preston J (2014) Exploring areas of consensus and conflict around values underpinning public involvement in health and social care research: a modified Delphi study. BMJ Open 4(1): e004217. 10.1136/bmjopen-2013-004217 PMC3902382 24413356

[bibr64-1744629517723485] Snow R Crocker JC Crowe S (2015) Missed opportunities for impact in patient and carer involvement: a mixed methods case study of research priority setting. Research Involvement and Engagement 1(1): 1–13. 29062496 10.1186/s40900-015-0007-6 PMC5611607

[bibr65-1744629517723485] Staley K (2009) Exploring Impact: Public Involvement in NHS, Public Health and Social Care Research. Eastleigh: INVOLVE.

[bibr66-1744629517723485] Stalker K (1998) Some ethical and methodological issues in research with people with learning difficulties. Disability & Society 13: 5–19.

[bibr67-1744629517723485] Staniszewska S Denegri S (2013) Patient and public involvement in research: future challenges. Evidence Based Nursing 16(3): 69. 23749800 10.1136/eb-2013-101406

[bibr68-1744629517723485] Szmukler G Staley K Kabir T (2011) Service user involvement in research. Asia-Pacific Psychiatry 3(4): 180–186.

[bibr69-1744629517723485] Tassé MJ Schalock R Thompson J (2005) Guidelines for interviewing people with disabilities: Supports Intensity Scale. Washington DC: American Association on Intellectual and Developmental disabilities.

[bibr70-1744629517723485] Thompson J Bissell P Cooper CL (2014) Exploring the impact of patient and public involvement in a cancer research setting. Qualitative Health Research 24(1): 46–54. 24277776 10.1177/1049732313514482 PMC4509885

[bibr71-1744629517723485] Tritter J McCallum A (2006) The snakes and ladders of user involvement: moving beyond arnstein. Health Policy 76(2): 156–168. 16006004 10.1016/j.healthpol.2005.05.008

[bibr72-1744629517723485] Tritter JQ (2009) Revolution or evolution: The challenges of conceptualizing patient and public involvement in a consumerist world. Health Expectations 12(3): 275–287. 19754691 10.1111/j.1369-7625.2009.00564.x PMC5060496

[bibr73-1744629517723485] Tuffrey-Wijne I Bernal J Hollins S (2008) Doing research on people with learning disabilities, cancer and dying: ethics, possibilities and pitfalls. British Journal of Learning Disabilities 36(3): 185–190.

[bibr74-1744629517723485] Tyrer F Dunkley AJ Spong R (2016) Involving service users with intellectual disability in research: experiences from the STOP diabetes study. Journal of Policy and Practice in Intellectual Disabilities.

[bibr75-1744629517723485] Walmsley J (2001) Normalisation, emancipatory research and learning disability. Disability and Society 16(2): 187–205.

[bibr76-1744629517723485] Walmsley J (2004) Involving users with learning difficulties in health improvement: lessons from inclusive learning disability research. Nursing Inquiry 11(1): 54–64. 14962347 10.1111/j.1440-1800.2004.00197.x

[bibr77-1744629517723485] Walmsley J Johnson K (2003) Inclusive Research with People with Learning Disabilities: Past, Present and Future. London, UK: Jessica Kingsley.

[bibr78-1744629517723485] Whitehurst T (2007) Liberating silent voices – perspectives of children with profound & complex learning needs on inclusion. British Journal of Learning Disabilities 35(1): 55–61.

[bibr79-1744629517723485] Wright D Foster C Amir Z (2010) Critical appraisal guidelines for assessing the quality and impact of user involvement in research. Health Expectations 13(4): 359–368. 20629767 10.1111/j.1369-7625.2010.00607.x PMC5060547

[bibr80-1744629517723485] Zarb G (1992) On the road to damascus: first steps towards changing the relations of disability research production. Disability, Handicap & Society 7(2): 125–138.

